# Influenza-Like Illness Sentinel Surveillance in Peru

**DOI:** 10.1371/journal.pone.0006118

**Published:** 2009-07-01

**Authors:** V. Alberto Laguna-Torres, Jorge Gómez, Víctor Ocaña, Patricia Aguilar, Tatiana Saldarriaga, Edward Chavez, Juan Perez, Hernán Zamalloa, Brett Forshey, Irmia Paz, Elizabeth Gomez, Roel Ore, Gloria Chauca, Ernesto Ortiz, Manuel Villaran, Stalin Vilcarromero, Claudio Rocha, Omayra Chincha, Gerardo Jiménez, Miguel Villanueva, Edwar Pozo, Jackeline Aspajo, Tadeusz Kochel

**Affiliations:** 1 US Naval Medical Research Center Detachment, Lima, Peru; 2 Dirección General de Epidemiología del Ministerio de Salud del Perú, Lima, Peru; 3 Dirección Regional de Salud de Piura Ministerio de Salud del Perú, Piura, Peru; 4 Centro Medico Militar Sullana, Piura, Peru; 5 Universidad Nacional de San Agustín, Arequipa, Peru; 6 Universidad Nacional de Ucayali, Pucallpa, Peru; 7 Dirección Regional de Salud de Puno, Ministerio de Salud del Perú, Puno, Peru; University of Georgia, United States of America

## Abstract

**Background:**

Acute respiratory illnesses and influenza-like illnesses (ILI) are a significant source of morbidity and mortality worldwide. Despite the public health importance, little is known about the etiology of these acute respiratory illnesses in many regions of South America. In 2006, the Peruvian Ministry of Health (MoH) and the US Naval Medical Research Center Detachment (NMRCD) initiated a collaboration to characterize the viral agents associated with ILI and to describe the clinical and epidemiological presentation of the affected population.

**Methodology/Principal Findings:**

Patients with ILI (fever ≥38°C and cough or sore throat) were evaluated in clinics and hospitals in 13 Peruvian cities representative of the four main regions of the country. Nasal and oropharyngeal swabs, as well as epidemiological and demographic data, were collected from each patient. During the two years of this study (June 2006 through May 2008), a total of 6,835 patients, with a median age of 13 years, were recruited from 31 clinics and hospitals; 6,308 were enrolled by regular passive surveillance and 527 were enrolled as part of outbreak investigations. At least one respiratory virus was isolated from the specimens of 2,688 (42.6%) patients, with etiologies varying by age and geographical region. Overall the most common viral agents isolated were influenza A virus (25.1%), influenza B virus (9.7%), parainfluenza viruses 1, 2, and 3, (HPIV-1,-2,-3; 3.2%), herpes simplex virus (HSV; 2.6%), and adenoviruses (1.8%). Genetic analyses of influenza virus isolates demonstrated that three lineages of influenza A H1N1, one lineage of influenza A H3N2, and two lineages of influenza B were circulating in Peru during the course of this study.

**Conclusions:**

To our knowledge this is the most comprehensive study to date of the etiologic agents associated with ILI in Peru. These results demonstrate that a wide range of respiratory pathogens are circulating in Peru and this fact needs to be considered by clinicians when treating patients reporting with ILI. Furthermore, these data have implications for influenza vaccine design and implementation in South America.

## Introduction

Influenza-like illnesses (ILI) are a significant source of morbidity and mortality worldwide. In many parts of the world, particularly in temperate regions of the Northern Hemisphere such as the United States and Europe, the etiologic agents associated with ILI have been well characterized. In much of Latin America, however, the epidemiology and etiology of ILI are poorly understood. ILI can be attributed to a wide range of respiratory viruses, including influenza viruses, adenoviruses, respiratory syncytial virus (RSV), enteroviruses, human metapneumovirus (HMPV), and parainfluenza viruses. Adenovirus, RSV, and parainfluenza viruses can cause severe disease particularly in children, accounting for a considerable proportion of childhood morbidity and mortality [1], [2]. Enteroviruses and HSV have been isolated from patients with acute respiratory infections and these viruses may also cause infections such as pharyngitis [3], [4]. Patients infected by these diverse viral pathogens present with widely overlapping symptomology, which render clinical diagnosis unreliable and severely limits etio-epidemiological studies.

The predominant pathogens of ILI are typically the influenza viruses which cause annual recurrent epidemics affecting an estimated 5–15% of the population presenting with upper respiratory tract infections worldwide. The World Health Organization (WHO) estimates that globally there are 3–5 million severe cases and 250,000–500,000 deaths globally [5] due to influenza every year, with most deaths occurring among elderly populations. Influenza viruses are genetically labile and are thus able to adapt and elude the host immune response, leading to regular seasonal influenza circulation and occasional pandemic events [5]–[7]. Vaccination is considered the best transmission prevention method; however, the vaccine must be updated annually to match the circulating influenza strains [8]. Through an international influenza surveillance network, the WHO identifies the viral strains that are to be included in the vaccine for the following transmission period in both the Northern and Southern Hemispheres [9]–[11]. Selecting the appropriate antigens for the annual vaccine requires surveillance systems to be in place worldwide to monitor circulating variants.

Since 1998, the Ministry of Health (MoH) of Peru has conducted virological surveillance of influenza and other respiratory viruses [12], [13]. In 2006, to strengthen the National Peruvian surveillance program, the MoH Sub-committee of Influenza Surveillance invited the US Naval Medical Research Center Detachment (NMRCD) in Lima, Peru to assist in increasing surveillance coverage by establishing new sentinel sites. Since then, NMRCD has augmented the existing program with support for obtaining and processing samples from new sentinel sites as well as providing data to the Epidemiology Directorate and the National Institute of Health of Peru. The objectives of the present study were to describe the clinical and epidemiological presentation of ILI in the study population, identify the etiologic agents associated with ILI, and molecularly characterize the influenza viruses isolated from patient specimens. Herein we present the data on the viral agents causing ILI in Peru from June 2006 through May 2008, based on results from NMRCD-MoH study sites in 13 cities across the country.

## Materials and Methods

### Case Definition

At each site, trained medical personnel were responsible for properly identifying and classifying patients with ILI. The case definition was “any person with a sudden onset of fever (≥38°C) and cough or sore throat fewer than 5 days in duration, accompanied or not by general symptoms such as myalgias, prostration, headache or malaise” [14].

### Study Population

The study population included every patient with ILI, regardless of age, who sought attention in participating health centers between June 2006 and May 2008 and agreed to participate in the study.

**Figure 1:**
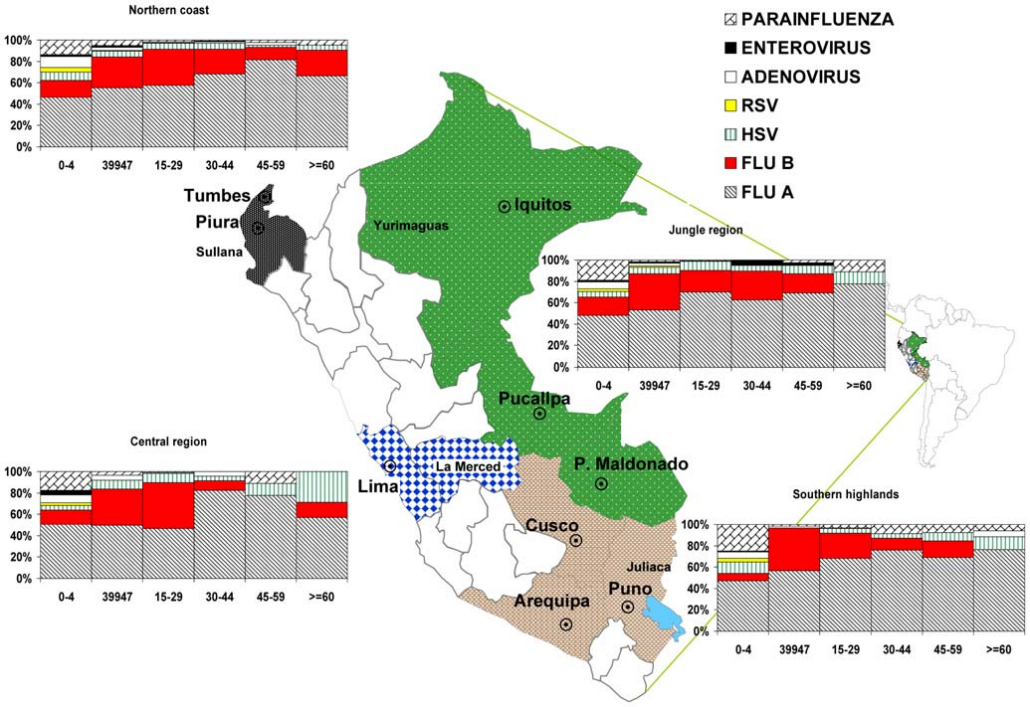
Viral etiology of ILI in Peru distributed by age and geographic region, June 2006–May 2008.

Peru is divided by the Andes Mountains into naturally distinct regions (highlands, coastal desert and jungle) all extending the entire length of the country. The capital city of Lima serves as a reference point dividing the northern and southern regions. Participants (outpatient or inpatient) were recruited in 31 hospitals or health centers in 13 Peruvian cities located in 10 provinces, which were intended to represent four distinct geographic regions of the country. The sites included in this study were from 1) the southern highlands, including Arequipa, Cusco, Puno and Juliaca, all located over 2,500 meters above sea level; 2) the northern coastal desert, including Tumbes, Piura and Sullana, all with limited rainfall (<20 cm/year); 3) the jungle region, including Puerto Maldonado, Pucallpa, Iquitos and Yurimaguas, where rainfall exceeds 200 cm yearly; and 4) the central region of the country, including La Merced (Chanchamayo) and Lima (Figure 1). In each city, at least two accessible health centers were included from urban areas. Hospitalization was noted if the patient spent at least one night in the hospital or health center.

Study participants from Sullana, Arequipa, Puno, Pucallpa and Iquitos also included military personnel who presented at local military health centers. Military populations were included from ILI outbreaks occurring in any military base (army or navy) within Peru. While participants recruited during outbreak investigations were excluded from epidemiological analyses of each geographical region, some outbreak-related samples were included in the molecular characterization to provide broader coverage and description of circulating influenza virus variants.

### Data Collection and Management

Data on gender, age, lost work or school days, previous treatment, medical attention before enrollment, influenza vaccination status and travel in the last 7 days were collected utilizing a case report form (CRF) from all participants who met the case definition.

Temporal distribution of the results were recorded by month and epidemiological week (EW) during the study period, taking into account the number of ILI cases identified and the number of confirmed cases of influenza A and B in each geographical region. Weekly reports of enrolled ILI participants and monthly laboratory results were sent to the MoH. Regular personnel training in protocol procedures and semi-annual site visits were conducted as part of the strategy to improve sampling, storage and shipping procedures.

Local coordinators notified NMRCD when an outbreak was suspected, and additional supplies were shipped to the site. When an increased number of cases were detected in any health center, Peruvian officers were notified and the Regional MoH Epidemiology Directorate determined if an outbreak investigation was necessary.

### Laboratory Aanalysis

#### Sample collection

Two types of samples were obtained from the cases for diagnostic testing: a nasal swab for the Rapid Influenza Test (RIT; QUICKVUE Influenza test, Quidel, San Diego, CA) and an oropharyngeal swab for viral isolation. The RIT was processed on site, and the results were provided to the patient. Oropharyngeal swabs were placed in transport media and stored at −70°C until they were delivered on dry ice to NMRCD for laboratory analysis.

#### Virus isolation and identification

Patient specimens were inoculated into four cell lines for virus isolation: Madin-Darby canine kidney (MDCK), African green monkey kidney (Vero76 and VeroE6) and Rhesus monkey kidney (LLCMK2) cells. Upon the appearance of a cytopathic effect (CPE) or after ten (or thirteen in the case of Vero cells) days of culture, the cells were spotted onto microscope slides. Cell suspensions were dried and fixed in chilled acetone for 15 minutes. Immunofluorescence assay (IFA) was performed to identify the virus isolates using a direct fluorescense assay (DFA). The Respiratory Virus Screening and Identification Kit (D3 DFA Respiratory Virus Diagnostic Hybrids, Athens, OH) was utilized for the identification of adenoviruses, influenza A virus, influenza B virus, parainfluenza viruses (types 1, 2, and 3), and RSV. The D3 DFA HSV identification kit and the D3 IFA Enterovirus ID kit (Diagnostic hybrids) were utilized for the identification of HSV and enteroviruses respectively. All assays were performed following the manufacturer's established protocols.

Cases with positive RIT results, but without confirmatory virus isolation results were further tested for influenza A and B viruses by using a one step reverse transcriptase-polymerase chain reaction (RT-PCR) with the influenza primers described below. The viral etiology of cases was based on the isolation of virus (CPE and/or IFA/DFA positive) and/or a positive result by both RIT and RT-PCR

#### RNA extraction and RT-PCR

For the genetic analyses of influenza viruses, viral RNA extraction was performed from the supernatant of infected MDCK cells using a QIAamp Viral RNA kit (QIAGEN, Valencia, CA) following the manufacturer's protocol. The one-step RT-PCR was performed with primers that amplify the hemagglutinin (HA) gene of influenza A and influenza B viruses using the SuperScript III One-Step RT-PCR System kit (Invitrogen, San Diego, CA). The following primers were used for the amplification of H1 influenza A viruses: H1F-6 (5′-AAGCAGGGGAAAATAAAA-3′) and H1R-1193 (5′-GTAATCCCGTTAATGGCA-3′); for H3 influenza A viruses: H3F-7 (5′-ACTATCATTGCTTTGAGC-3′) and H3R-1184 (5′-ATGGCTGCTTGAGTGCTT-3′); for influenza B viruses: BHAF-36 (5′-GAAGGCAATAATTGTACT-3′) and BHAR-1140 (5′-ACCAGCAATAGCTCCGAA-3′). Five µl of the extracted RNA was added to 20 µl of master mix containing the enzyme mixture (SuperScript III RT/Platinum Taq), 2X reaction mixture (containing 0.4 mM of each dNTP and 3.2 mM of MgSO_4_) and 20 µM of each primer. Cycling conditions included a reverse transcription step at 50°C for 30 minutes and a denaturation step at 94°C for 2 min. Cycling conditions of the PCR were 40 cycles of 94°C for 15 seconds, 52°C for 30 seconds, and 68°C for 75 seconds, followed by a final incubation step at 68°C for 5 min.

The RT-PCR products were purified using Centri-Sep Columns (Princeton Separation, Englishtown, NJ) and sequenced using the BigDye Terminator v. 3.1 Cycle Sequencing Kit (Applied Biosystems, Foster City, CA) following manufacturers' standard procedures. Sequences were analyzed and edited using the Sequencer 4.8 software (Applied Biosystems Foster City, CA)

#### Sequencing and phylogenetic analysis

The nucleotide sequences were aligned using the Clustal program in the Mac Vectorsoftware package (Mac Vector Inc., Cary, NC), and phylogenetic analyses were performed using the neighbor joining and maximum likelihood algorithms implemented in the phylogenetic analysis using parsimony (PAUP) software [15], [16]. For the neighbor joining analyses the HKY85 distance was used and bootstrap values were calculated based on 1000 replicates to place confidence values on grouping within trees.

### Ethical Management

This surveillance protocol (NMRCD.2002.0019) was approved as less than minimal risk research by the NMRCD Institutional Review Board (IRB) and the Peruvian MoH, and written consent forms were not required. Stamped, approved information sheets were used in place of written consent forms. Additionally, the study database was shared with the Epidemiology Directorate at the Peruvian MoH

### Statistical Analysis

The clinical-epidemiological forms were entered into a database created in Microsoft (MS) Office Access 2003, and data were analyzed using dynamic tables in MS Excel 2003.

The Chi square and Fisher exact tests were used to compare means and associations using SPSS software version 10.0 (SPSS Inc., Chicago, IL).

## Results

### General Findings

A total of 6,835 participants from general hospitals and health centers located in 13 different cities were enrolled in this study, of whom 3,786 (55%) were male (Table 1). Of those, participants, 6,308 were recruited during routine surveillance activities, and 527 were recruited during outbreak investigations. The patients ranged in age from ≤1 to 94 years with a median age of 13 years and a mean age of 16 years (SD 16.4 yrs); 56.4% of participants were under 15 years of age, and 8.1% were older than 45 years. From 4,552 ILI cases between 5 and 65 years of age, 1,213 (26.6%) reported lost school or working days because of the illness; 85% of whom lost 2 days or fewer (mean 1.7 days; SD 1.2 days). A total of 116 (1.7%) participants reported a history of influenza vaccination for that season (Table 1).

**Table 1:** Characteristics of the study population.

		No. (%)
**Number of samples (total patients enrolled)**		6835(100)
**Respiratory virus positive (regular passive surveillance)**		2688(42.6)
**Influenza virus positive (influenza A and B)**		2192(34.8)
**Respiratory virus positive (total patients enrolled)**		2840(41.6)
**Sex**	Female	3049(44.6)
	Male	3786(55.4)
**Age (N = 6385)**	Mean, ±SD	16, ±16.4 yrs
	Median, [range]	13, [0–94]
	0–4 yrs	2267(35.5)
	5–14 yrs	1337(20.9)
	15–29 yrs	1961(30.7)
	30–44 yrs	733(11.5)
	45–59 yrs	367(5.7)
	>60 yrs	154(2.4)
**Travel (last 7 days)**		888(13)
**Vaccination history**		116(1.7)
**Hospitalized**		209(3.1)
**Lost work/school days (n = 1213 between 5–65 years)**	Mean, ±Std	1.7, ±1.2 days
	Median, [range]	1, [0.13–12]
	<1 days	64(5.3)
	1 day	672(55.4)
	2 days	283(23.3)
	3 days	114(9.4)
	>4 days	80(6.6)
**Medical attention before enrollment**		2191(32.1)
**Previous treatment**	Antibiotics	1021(14.9)
	Others	2869(42)
	Unknown	160(2.3)
	No treatment	2508(36.7)
	Missing	277(4.1)
**Military population**		968(14.2)
**Civilian population**		5867(85.4)
**Outbreak population**		527(7.7)
**Positive rapid test**	Influenza A	1212(17.7)
	Influenza B	516(7.5)
	Undifferentiated	175(2.6)
	Negative rapid test	4592(67.2)
	No test	340(5)

### Laboratory Findings

Of the samples collected during routine surveillance, 2,688 (42.6%) were positive for respiratory viruses by isolation; of those 1,583 (25.1%) were positive for influenza A virus, 609 (9.7%) for influenza B virus, 115 (1.8%) for an adenovirus and 202 (3.2%) for parainfluenza 1, 2 or 3 viruses (Table 2). Viral etiology of ILI cases by age and region is shown in Figure 1.

**Table 2:** Viral etiology of Influenza-like Illness cases by Region. Peru. June 2006–May 2008.

			Total	South Highlands			Northern Coast		Jungle region			Central region	
			Count (%)	Arequipa	Cusco	Puno^1^	Tumbes	Piura^2^	Puerto Maldonado	Pucallpa	Iquitos^3^	La Merced	Lima
**Total**			**6308 (100)**	**309**	**459**	**274**	**940**	**1972**	**216**	**389**	**1178**	**279**	**292**
				**(%)**	**(%)**	**(%)**	**(%)**	**(%)**	**(%)**	**(%)**	**(%)**	**(%)**	**(%)**
**Total positives samples**			**2688 (42.6)**	**38.2**	**39.7**	**39.8**	**41.3**	**50.8**	**31**	**27.8**	**42.2**	**44.8**	**32**
**Results positives:**	Flu A		1583 (25.1)	22.3	27.7	25.5	25.9	27.8	19.0	17.5	23.9	25.4	21.9
		H1N1	102 (6.4)	1.4	2.4	8.6	8.6	3.1	9.8	8.8	8.5	18.3	10.9
		H3N2	130 (8.2)	18.8	18.9	15.7	2.9	8.2	19.5	4.4	3.9	1.4	10.9
	Flu B		609 (9.7)	6.1	9.2	7.3	5.7	12.9	5.6	3.9	13.0	11.1	2.7
	PARA FLU 1,2 & 3		202 (3.2)	6.8	1.5	2.9	3.2	4.2	3.2	3.3	1.5	3.9	1.7
	HSV		164 (2.6)	2.9	1.3	2.2	3.6	2.7	1.4	1.5	2.5	3.2	2.7
	Adenovirus		115 (1.8)	1.0	0.2	2.2	3.2	2.4	0.5	0.8	1.2	1.8	1.7
	RSV		40 (0.6)			1.1	0.3	1.3	1.4	0.3	0.2		0.7
	Enterovirus		31 (0.5)	0.3			0.4	0.7	0.5	0.8	0.5		1.0
	Others		6 (0.1)				0.1	0.2			0.1		
**Total negative samples**			**3620 (57.4)**	**61.8**	**60.3**	**60.2**	**58.7**	**49.2**	**69.0**	**72.2**	**57.8**	**55.2**	**68.5**
			**Total**	**South Highlands**			**Northern Coast**		**Jungle region**			**Central region**	
**Co-Infeccions**	**Total Co-Infeccions**		**62**	**9**			**37**		**11**			**5**	
	Most frequent co-infection: Flu A-HSV		20	3			9		4			4	
	Most frequent agent: Flu A		39	6			24		4			5	
			**Total**	**South Highlands**			**Northern Coast**		**Jungle region**			**Central region**	
			Count	Arequipa	Cusco	Puno	Tumbes	Piura	Puerto Maldonado	Pucallpa	Iquitos	La Merced	Lima
**Total Outbreak**			**527 (100)**	**0**	**0**	**0**	**267**	**79**	**0**	**0**	**0**	**82**	**99**
				**(%)**	**(%)**	**(%)**	**(%)**	**(%)**	**(%)**	**(%)**	**(%)**	**(%)**	**(%)**
**Positive samples**			**152 (28.8)**				**26.6**	**16.5**				**58.5**	**20.2**
	Flu A		43 (8.2)				4.5	8.9				19.5	8.1
		H3N2	4 (9.3)				25.0						12.5
	Flu B		99 (18.8)				21.0	2.5				39.0	9.1
	HSV		12 (2.3)				1.5	5.1					4.0
**Negative samples**			**375 (71.2)**				**73.4**	**83.5**				**41.5**	**79.8**

1 “Puno” includes data from Puno and Juliaca cities.

2 Piura includes data from Piura and Sullana cities.

3 “Iquitos” includes data from Iquitos and Yurimaguas cities.

Among sites (Table 2), the percentage of samples positive for one or more viral pathogens ranged between 27.8% (Pucallpa) and 50.8% (Piura). The highest prevalence of influenza B virusamong ILI patients was in La Merced, Iquitos and Piura, all exceeding 11% of total cases (Table 2), which was significantly higher than in the other locations (p<0.001). Relative to the other study sites, a significantly higher prevalence of adenovirus infection was observed among study participants in the northern coastal sites of Tumbes and Piura (p<0.001) and a significantly lower prevalence of adenovirus infection was observed at study sites in Cusco (p<0.001). HSV was isolated from 164 (2.6%) ILI patients and the highest percentage of HSV isolation was observed in the coastal cities of Lima, Tumbes, and Piura (Table 2). Viral co-infection was detected in 34 (20.7%) HSV-positive cases, including 20 (12%) who had HSV and influenza A virus co-infection (Table 2), which was the most frequent co-infection found in the study. We also identified 3 (0.04%) patients with co-infection by HSV and parainfluenza viruses. Of patients with HSV infection, 106 (60%) were under 15 years of age, 66 (38%) of whom were between 0–4 years of age. In total, RSV was isolated from 40 (0.6%) cases; however it was not isolated from patients reporting to clinics in Arequipa, Cusco or La Merced.

The prevalences of influenza A and B viruses were significantly different between the different age groups (p<0.001), with a higher prevalence among patients older than 5 years. Parainfluenza viruses, enteroviruses, RSV, HSV and adenoviruses were more common among patients younger than 5 than in the older age groups (Figure 1).

To determine the efficacy of in situ influenza testing, RIT results were compared with laboratory isolation results. Sensitivity of RIT was 73% (1,636/2,250) and specificity was 94% (3,978/4,245). The positive predictive value of the RIT was 85% (1,636/1,903).

### Clinical Data

Fever, cough, malaise and rhinorrhea were the major symptoms (each observed in over 80% of the patients) for all age groups and geographical regions, although sore throat (71%) and headache (63%) were also frequently recorded. Rhonchus (17%) and wheezing (7%) were more frequent in patients younger than 5 years in contrast to what was seen in older patients, especially those who were positive for an influenza virus. A total of 209 (3.1%) patients with ILI were hospitalized (Table 1). There was no significant association between any pathogen and higher hospitalization rates (p>0.05).

Sore throat and vesicular lesions on the lips, mouth or throat were reported by 120 (68%) and 11 (6.2%) of the HSV-positive patients, respectively.

A total of 1,021 (14.9%) ILI patients reported receiving antibiotics before enrollment into the study. Of these patients, 248 (24%) were found to be infected with influenza A virus, 121 (11.9%) with influenza B virus, 18 (1.7%) with adenoviruses and 30 (2.9%) with parainfluenza viruses. RIT was positive for 32.7% of participants who had received antibiotics at some point prior to enrollment.

### Outbreaks and Military Population

A total of 968 military personnel were enrolled, making up 14% of the total study population (Table 1). Of those, 466 (48%) were enrolled during regular surveillance activities maintained at regional military hospitals at Sullana, Arequipa, Pucallpa, and Iquitos. The remaining 502 (52%) were enrolled during outbreaks in military populations at Tumbes, Piura, La Merced and Lima, making up the majority (95%) of the 527 total participants recruited during outbreak investigations (Table 1). One of the main outbreaks in 2007 included 124 ILI cases from two army bases in Tumbes on the northern coast[17]. A total of 66 influenza cases were confirmed by cell culture, including 54 cases of influenza B virus infection and 12 cases of influenza A virus infection. Isolates were genetically similar to the influenza B virus strain B/Texas/4/2006 (Victoria lineage) and the influenza H3N2 A virus strain A/Texas/91/2007 (A/Brisbane/10/07-lineage). In 2007 as well, a total of 82 ILI cases were enrolled during another outbreak at the La Merced army base in central Peru; 32 were influenza B virus-positive by isolation and 16 were influenza A virus-positive (Table 2).

### Temporal and Geographical Distribution of Influenza A and B Virus Infections

**Figure 2:**
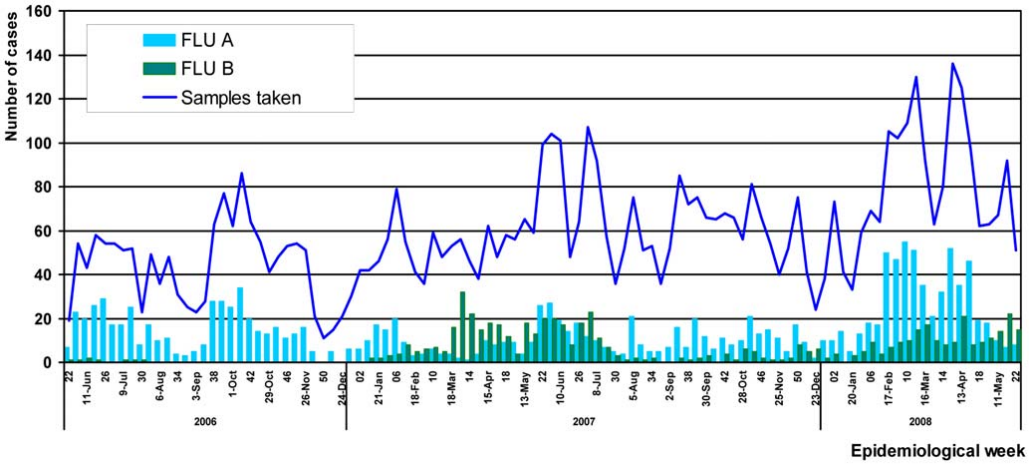
Temporal distribution by month and epidemiological week (EW) of influenza A and B confirmed cases, Peru 2006–2008.

**Figure 3:**
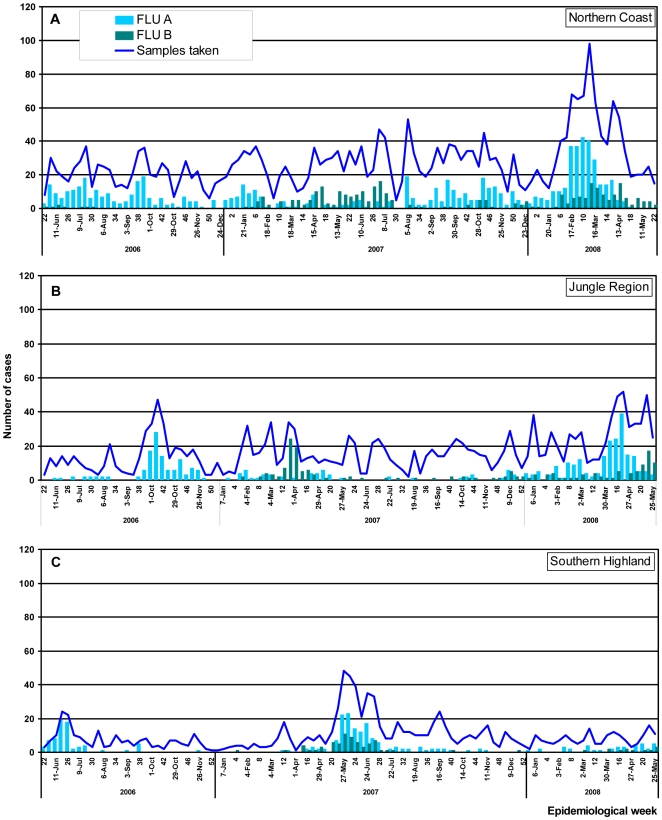
Temporal distribution of total ILI and influenza A and B cases by month and epidemiological week (EW) and by region. A)northern coast region. B) jungle regionC) south highlands region.

The temporal distribution of total ILI and confirmed influenza cases identified at study sites nationwide between the 22^nd^ Epidemiological week (EW) of 2006 and the 20^th^ EW of 2008 is shown in Figure 2. During the second half of 2006, influenza A virus was predominant, while very few cases of influenza B virus were detected. All influenza B virus infections during 2006 were detected in study sites along the northern coast (Figures 3A, 3B, 3C,). Influenza B virus continued to circulate at low levels between the 2^nd^ and 10^th^ EW of 2007 before becoming the predominant influenza virus species during the 10^th^ and 28^th^ EW (Figure 2). A simultaneous increase in the healthcare demand for ILI cases in Tumbes, Piura, Arequipa, Iquitos and La Merced was observed during this period.

In the southern highlands, peaks of influenza A and B virus infections were observed between the19th and the 28th EW in 2007 coinciding with the winter season in that region. With the exception of this time period the number of influenza A and B cases were relatively low in the southern highlands during 2007.

In the northern coast region (Tumbes and Piura), influenza virus circulation was detected throughout the year during 2007; influenza B viruses were identified mainly between the 14^th^ and the 26^th^ EW. This detection of influenza B viruses corresponds to an outbreak identified in two army bases in Tumbes (EW 16 through 18). In the Amazon jungle region (Iquitos, Pucallpa Yurimaguas and Puerto Maldonado), very few cases of influenza A virus infection were detected during 2007, although an increase of influenza B cases was detected between the 5^th^ and the 16^th^ EW.

Over the period of 2008 reported here (through EW 22), influenza A viruses predominated throughout the country, approaching three times the number of influenza B cases (Figure 2). In study sites in the southern highlands region very few cases of influenza A and or B were detected during the first months of 2008 (1^st^–22^nd^ EW). Comparatively, in the northern coast region, influenza A cases were more common than influenza B cases, although an increase number of influenza B cases were observed between the 6^th^ and 16^th^ EW (Figures 3A and 3C). In the jungle region influenza B cases were also identified almost weekly during the 1^st^–22^nd^ EW period (Figure 3B).

### Phylogenetic Analysis of the Influenza isolates

#### H1N1 influenza A viruses

**Figure 4:**
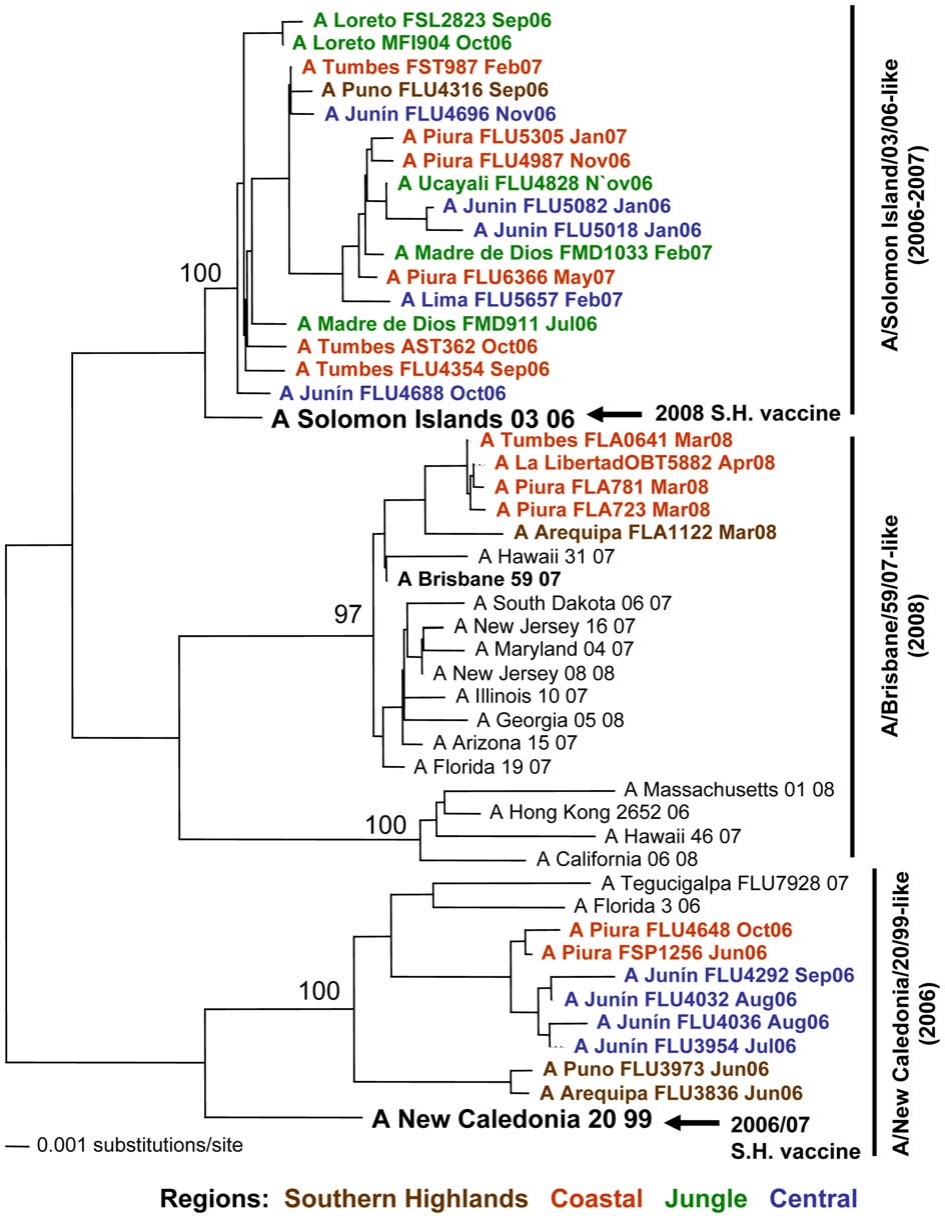
Phylogenetic tree based on the partial hemagglutinin (HA) sequence of influenza A H1N1 viruses. Numbers indicate bootstrap values. The legend indicates the geographical origin of the strains: southern highlands (brown), coastal (red), jungle (green) and central (blue) regions. Arrows indicate the recommended vaccine strain for the Southern Hemisphere for each year of the study period.

Genetic analyses based on the HA gene of 102 H1N1 influenza A isolates from Peru revealed three distinct genotypes: 1) A/Solomon Islands/03/06-like, 2) A/Brisbane/59/07-like and, 3) A/New Caledonia/20/99-like (Figure 4).

Among the 2006 isolates from the northern coast (in red), central (in blue) and southern highlands (in brown) regions, two distinct genotypes were observed: A/Solomon Island/03/06-like and the A/New Caledonia/20/99-like. The latter also includes the recommended 2006/2007 vaccine strain for the Northern and Southern Hemispheres (A/New Caledonia/20/99). All isolates from the jungle region during 2006 grouped within the A/Solomon Islands/03/06-like genotype.

In 2007, the A/Solomon Islands/03/06-like genotype continued to circulate in Peru. This genotype does not include the recommended 2007 vaccine strain for the Southern Hemisphere (A/New Caledonia/20/99).

The circulation of the A/Brisbane/59/07-like genotype was detected among the 2008 isolates from the northern coast and southern highland regions. Furthermore, this genotype does not include the recommended 2008 vaccine strain A/Solomon Islands/03/06.

#### H3N2 influenza A viruses

**Figure 5:**
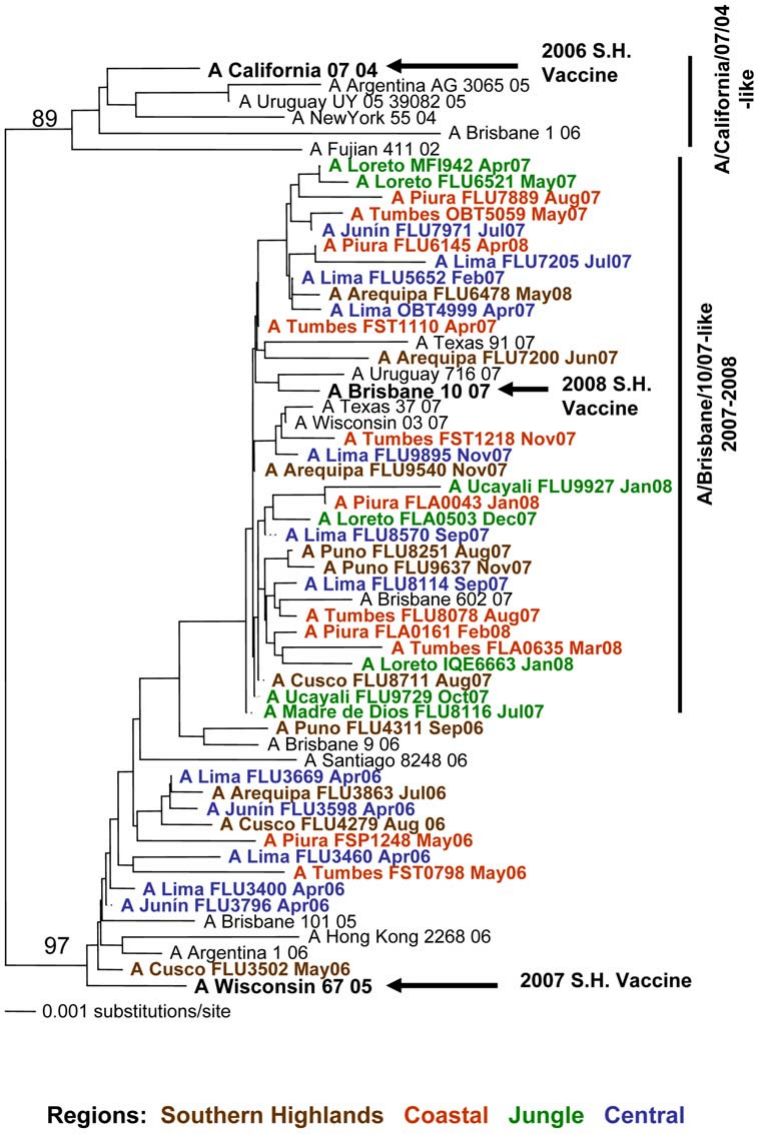
Phylogenetic tree based on the partial hemagglutinin (HA) sequence of influenza A H3N2 viruses. Numbers indicate bootstrap values. The legend indicates the geographical origin of the strains: southern highlands (brown), coastal (red), jungle (green) and central (blue) regions. Arrows indicate the recommended vaccine strain for the Southern Hemisphere for each year of the study period.

Genetic analyses based on the HA gene of 134 H3N2 influenza A viral isolates circulating in Peru from 2006 through 2008 revealed that the 2007–2008 influenza strains grouped within the A/Brisbane/10/07-like genotype, which includes the 2008 vaccine strain from the Southern Hemisphere (A/Brisbane/10/07). The influenza A H3N2 virus strains from all geographical regions of Peru were grouped into this single genotype (Figure 5). The 2006 influenza A H3N2 virus isolates differ genetically from the recommended 2006 vaccine strain A/California/07/04.

#### Influenza B viruses

**Figure 6:**
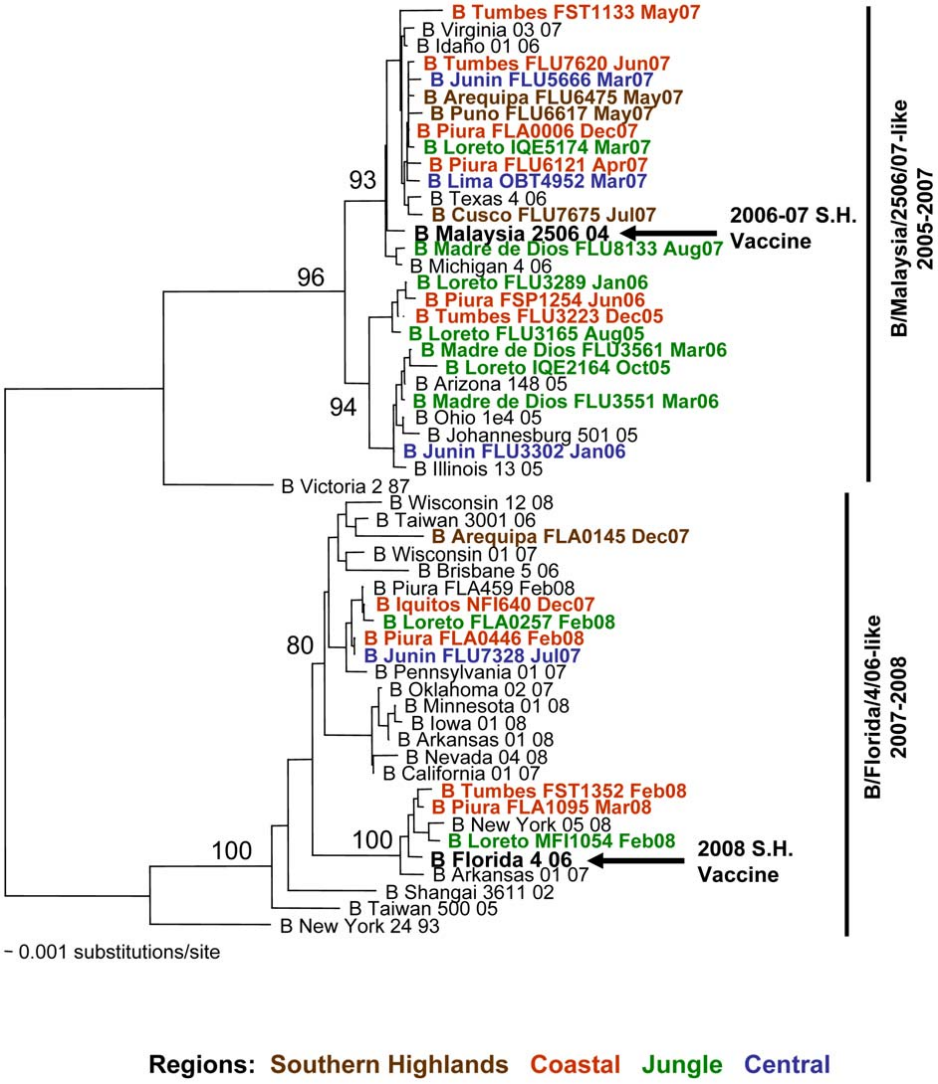
Phylogenetic tree based on the partial hemagglutinin (HA) sequence of influenza B viruses. Numbers indicate bootstrap values. The legend indicates the geographical origin of the strains: southern highland (brown), coastal (red), jungle (green) and central (blue) regions. Arrows indicate the recommended vaccine strain for the Southern Hemisphere for each year of the study period.

Phylogenetic analyses based on the HA sequence of 169 influenza B virus isolates revealed the presence of two genotypes in Peru: B/Malaysia/2506/07-like and B/Florida/4/06-like. These genotypes co-circulated in Peru in 2006 and 2007; however, the most recent influenza B virus isolates from 2008 belong to the B/Florida/04/06 genotype, which also includes the vaccine strain for the Southern Hemisphere (Figure 6).

## Discussion

Prior to this study, there has been little documentation on the circulating respiratory viruses in most regions of South America. To date, the data presented here represent the most thorough description of ILI-causing viruses throughout Peru. During the two years of surveillance reported in this study, upper respiratory viruses were isolated from greater than 40% of samples collected from nearly 7,000 patients with ILI. As expected, influenza A (23.8% of the total cases) was the predominant diagnosis. During the period of the study, however, we also observed the introduction and emergence of influenza B viruses in these study sites. In addition, parainfluenza viruses, enteroviruses, adenoviruses, and HSV were isolated from patient specimens, which collectively contributed to 8.8% of all ILI cases in our study. The prevalences of influenza A and B viruses were significantly higher in ILI patients older than 5 years of age (p<0.001), while other viruses such as parainfluenza, adenovirus, enterovirus, HSV and RSV were more prevalent among participants younger than 5 years. These results are consistent with studies in other regions of South America [18], [19]. Taken together, these data demonstrate that a wide range of viruses need to be considered during the diagnosis of human respiratory illness.

HSV is a viral agent not usually considered in patients with upper respiratory complaints. In our study HSV was isolated from 176 (2.6%) of ILI patients. In addition to the respiratory symptoms, we found that 11 (6.2%) HSV-positive participants also had vesicular lesions on the lips, mouth or throat. Mc Millan [3], in a 16-month study of U.S. college students, found that 5.7% of patients with sore throats had HSV infection and, of those 34% had vesicular lesions and 71% had pharyngeal erithema. In our study, in the majority of cases where HSV was isolated, no other viral infection was detected. However, as HSV is a latent virus that may be reactivated during infections with other pathogens, we cannot conclude that HSV is the causative agent for the infections reported in this study. In addition, it should be noted that nearly 60% of total ILI cases were undiagnosed; suggesting that in HSV-positive cases where no other infection was detected as-of-yet unidentified pathogens may be responsible for the ILI symptoms. A control group without ILI symptoms will be needed to draw more definite conclusions on the role of HSV as a causative agent of ILI symptoms. Nevertheless these data suggest that in the future, HSV should be considered in diagnostic evaluations as a potential cause of ILI.

Distribution of the ILI-causing viruses varied by study site and by region. For example, adenoviruses were more common along the northern coast, in Tumbes and Piura (p<0.001) and much less common in Cusco (p<0.001). The geographic distribution of influenza viruses varied considerably as well. For influenza A viruses, the H3N2 strains predominated in the southern regions of the country (Arequipa, Cusco, Puno, and Puerto Maldonado, specifically), while influenza B viruses were more common in the northern and central regions (Tumbes, Piura, Iquitos, and La Merced). However, it should be noted that almost all detected influenza strains and lineages were found in all study sites, suggesting that newly introduced viral strains will spread to all regions of the country. In addition, while two years is not sufficient time for an adequate description of the seasonal patterns of influenza transmission; we did observe trends in different regions. In the southern highlands, sunny days and dry weather are characteristic during the autumn and spring. It was during the winter season when temperatures fall to 3 to −5°C that an increased number of influenza A and B cases were detected in both 2006 and 2007. This is consistent with unpublished data from the MoH. Comparatively, we found another trend in the northern coast and jungle regions where high humidity and hot weather are characteristic of the entire year; in these regions influenza virus transmission was detected throughout the year.

One shortcoming of a sentinel surveillance program such as this is the potential for sampling bias. As we have limited our studies to two health centers in most cities, our results may not be representative of the entire population. Additionally, we are unable to calculate incidence rates based on our study due to this bias and the lack of reliable population data. To address this shortcoming, we are establishing community-based active surveillance programs in several of these sites to better quantify the impact of ILIs on the population at large. One advantage of the present surveillance is the ability to identify increases in the number of patients seeking medical attention due to ILI symptoms and the diagnosis of the viruses related to such increases, while requiring fewer resources than would be necessary in a population-based study. Furthermore, we found similar influenza viruses present throughout the country, suggesting that a sentinel surveillance program is sufficient for detecting and describing currently circulating influenza lineages.

One objective of this study was the identification of circulating influenza strains to help evaluate current vaccine components and inform the composition of future vaccine cocktails. The MoH is planning to start massive vaccination campaigns for at-risk populations in Peru. For influenza A H1N1 viruses, three genotypes were observed: A/Solomon Island/03/06-like, A/Brisbane/59/07-like and A/New Caledonia/20/99-like. These genotypes include isolates that circulated in Peru in 2006 but have not been detected since. It is unclear whether this genotype has become extinct or whether it continues to circulate at low levels in the Peruvian population. Importantly, our genetic analyses revealed that the recommended 2007 vaccine strain A/New Caledonia/20/99 does not group with the H1N1 strains that circulated in Peru in 2007, suggesting that the vaccine was not protective against the 2007 circulating strains. Similarly, the 2008 recommended vaccine strain A/Solomon Islands/03/06 does not group with the 2008 isolates from Peru.

In contrast to the genetic diversity observed with the influenza A H1N1viruses, influenza A H3N2 isolates circulating in Peru in 2007 and 2008 grouped into a single clade irrespective of geographical regions. These isolates were found to be genetically similar to the A/Brisbane/10/07 vaccine strain, supporting the selection of the strain as part of the 2008 vaccine for the Southern Hemisphere. Importantly, the 2006 isolates from Peru were found to be genetically distinct to the recommended vaccine strain A/California/07/04. Overall, these data highlight the necessity for continuous influenza surveillance in South America and the need for sharing this information with WHO reference laboratories for proper evaluation and better selection of future Southern Hemisphere vaccines.

Genetic analyses of influenza B virus isolates revealed the presence of two genotypes in Peru: B/Malaysia/2506/07-like and B/Florida/4/06-like. Both genotypes co-circulated in Peru in 2006 and 2007; however, the most recent influenza B isolates from 2008 belong to the B/Florida/4/06 genotype, which also includes the vaccine strain for the Southern Hemisphere (B/Florida/04/06). Future studies should identify whether this genotype will be the dominant genotype in the current influenza season or whether both lineages will continue to co-circulate. Such data will be important for defining the components of the yearly vaccine.

In Peru like in other South American countries [20], the unnecessary use of antibiotics is a generalized problem. In this study, 15% of the participants had received antibiotics prior to enrollment, and thus prior to a definitive diagnosis. A large percentage of these participants were found to have a viral agent associated with their illness. These data demonstrate that unnecessary antibiotic use is common for treatment of a patient with influenza-like illness, even without a definitive diagnosis of the etiologic agent [21]. For many of these participants the purchase of antibiotics combinated with paying for the clinic visit can be a major economic imposition thus, it is important to have a better understanding of the pathogens most commonly associated with ILI in the region for both diagnostic and economic purposes. In this study, RIT results were available at the time of enrollment, which helped decrease the unnecessary continual use of antibiotics and thus provided an additional economic benefit for the patients. Unfortunately our study design does not permit us to determine definitively what percentage of participants received antibiotics following a positive RIT result, as follow up interviews were not conducted. We also did not ask participants about use of antiviral drugs since viral inhibitors are not a commonly used treatment for influenza infection in Peru, mostly due to their high cost and low availability. It is interesting to note, nonetheless, that we found that a high percentage of circulating influenza A H3N2viruses are resistant to antiviral drugs such as Amantadine based on secondary analysis of these isolates [22].

These data provide initial background levels of transmission of influenza viruses and other ILI-related viruses in several regions of Peru. Such data are important for identifying strategic locations to establish cohorts in the context of intervention analyses and experimental vaccine efficacy trials. In addition these studies provide a springboard for future analyses of more severe complications of viral influenza, including primary influenza pneumonia and secondary bacterial pneumonia [23]. Future studies will also be focused on identifying the etiologic agents for the nearly 60% of ILI cases that remain undiagnosed. Retrospective analyses of these stored samples will be necessary to identify other circulating ILI-related viruses, including coronaviruses, HMPV and rhinoviruses, many of which can have severe disease outcomes particularly in young children and the elderly. In addition, bacterial pathogens are likely responsible for some of those undiagnosed cases and will need to be considered. We didn't test for bacterial infections and may have a role in some ILI cases either as primary or secondary infections.

Such further analyses are necessary to better understand the pathogens in circulation and associated with ILI in the region, as well as to define the relative disease burden imposed by each pathogen.
